# Visuospatial function in early Alzheimer’s disease: Preliminary study

**DOI:** 10.1590/S1980-57642009DN30300010

**Published:** 2009

**Authors:** Natália Bezerra Mota Quental, Sonia Maria Dozzi Brucki, Orlando Francisco Amodeo Bueno

**Affiliations:** 1 Post Graduate Student, Psychologist, Department of Psychobiology of the Federal University of São Paulo, São Paulo SP, Brazil; 2 PhD, Psychologist, Department of Psychobiology of the Federal University of São Paulo, São Paulo SP, Brazil; 3 MD, Neurologist, Department of Psychobiology of the Federal University of São Paulo, São Paulo SP, Brazil.

**Keywords:** Dementia, early Alzheimer disease, visuospatial function

## Abstract

Alzheimer’s disease (AD) is the most frequent cause of dementia, accounting for 55% of all cases. AD patients gradually lose functional capacity, manifesting deficits in attention, language, temporal and direction orientation, mood, socialization and visuospatial function. The visuospatial function entails identification of a stimulus and its location. AD patients can present deficits in visuo-spatial skills during initial stages of the disease, but in the course of clinical evolution this function can become severely impaired. One of the neuropsychological tests indicated to evaluate the visuospatial function is the VOSP (The Visual Object and Space Perception Battery).

**Objectives:**

The aim of this preliminary study was to detect visuospatial dysfunction in early AD patients using the VOSP, and assess its sensitivity in a Brazilian sample.

**Results:**

Controls outperformed AD patients on all neuropsychological evaluations, except the Corsi block tapping task and cancellation task-errors. The AD patients performed significantly worse on all object perception and two space perception (Number Location and Cube Analyses) subtests of the VOSP.

**Conclusion:**

The AD patients demonstrated impaired visuospatial function in several aspects. The subtests of the VOSP were found to be sensitive for detecting this impairment in mild cases.

## Abstract (pt)

A doença de Alzheimer (DA) é a causa mais frequente de demencia, atingindo 55% dos casos. Os pacientes com DA gradativamente perdem a capacidade funcional, apresentando comprometimento na atenção, na linguagem, na orientação temporal e espacial, uso de objetos, no humor, na socialização, na função visuoespacial. A função visuoespacial diz respeito a identificação de um estímulo e a sua localização. O paciente com DA pode apresentar perdas da habilidade visuo-espacial no início da doença, contudo no curso do quadro clínico esta função deve apresentar-se bastante comprometida. Um dos instrumentos utilizados para avaliar a função visuo-espacial é o VOSP (*Visual Object and Space Perception Battery*).

**Objetivos:**

A proposta deste estudo é avaliar as alterações na função visuoespacial em pacientes com DA de intensidade leve com a VOSP, e verificar a sensibilidade desta bateria em uma amostra brasileira.

**Resultados:**

Os controles obtiveram melhores resultados em todos os testes neuropsicológicos, com excessão do Blocos de Corsi e no teste de cancelamento - erros. Nos subtestes da VOSP os pacientes com DA mostraram uma significativa diferença em todos os subtestes de percepção de objeto e em dois de percepção de lugar (Localização de Número e Análise de Cubo).

**Conclusão:**

Os pacientes com DA leve mostraram ter a função visuoespacial comprometida em alguns aspectos. Os subtestes da VOSP mostraram-se sensiveis a esses déficits na fase leve da doença.

Dementia is a syndrome characterized by impairments of cognitive functions, including memory, language, abstraction, organization, projection, and visuospatial skills.^1^ In Brazil, according to Herrera,^2^ dementia prevalence is 7.1% among subjects aged older than 65 years. Incidence increases with advancing age at least until the age of ninety years. Dementia prevalence is also greatest among women and individuals with low educational level.^3^ Alzheimer’s disease (AD) is the most frequent cause of dementia, accounting for 55% of all cases in Brazil.^2^

In general, AD symptoms begin with a deficit in recent memory and executive functions. When the process advances, impairment spreads to other functions, such as semantic memory, language and visuospatial ability.^4^ The pathological hallmark of AD is the accumulation of neurofibrillary tangles and senile plaques, first in regions of the medial temporal lobe (transentorhinal and entorhinal cortex, hippocampus), and with progression of the disease these neuropathological alterations affect other neocortical areas.^5^

The visuospatial function involves identifying stimuli and their location. Studies indicate that the visuospatial tasks activate different cortical areas such as the V^5^ (Broadmann area) superior parietal lobule, parieto-occipital junction and premotor areas.^6-8^ Neuropsychological studies indicate that declines in visual sensory function in patients with early AD are more likely the result of cortical degenerative changes of AD (presence of neurofibrillary tangles and neuritic plaques in visual association cortex) rather than changes in the retina or retinocalcarine pathways. Therefore, visual dysfunction in AD involving attention, memory, motion and spatial cues may reflect pathology in these vision-related brain areas.^7,8^

In AD, the visuospatial function can be impaired in the beginning of the disease, and gradually declines with clinical evolution. Visuospatial deficits are common, manifesting in tasks that involve visual discrimination, analysis, spatial judgment, and perceptual organization.^5^ Generally in AD, the visuospatial deficits are not detected and go untreated because the patients have normal visual acuity.^9^ However, visuospatial problems can be found in a significant proportion of mild AD cases (20%) and are a key symptom of AD and other dementias.^10-12^ Some deficits in reading, numeric operations and orientation can be the result of visuospatial deficits and do not necessarily stem from impairment in memory or language, other symptoms commonly seen in AD.^9^ The evaluation of these deficits is necessary to provide more diagnostic information, and enable the development of new intervention approaches.

Currently, there are few reports evaluating visuospatial functions in the Brazilian literature. In a previous diagnostic consensus, authors suggested use of the “cookies theft description” and perception of embedded pictures as potential tools for detecting visuospatial compromise, but no Brazilian surveys have yet been performed.^13^ Most of the commonly used neuropsychological tests assessing visual and spatial perception rely on additional cognitive abilities.^14^ Some tests evaluate visual orientation, and consist of identifying object location in space. More complex instruments are available to assess spatial processing and use more complex activities such as drawing. Other instruments incorporate tasks that evaluate spatial perception, position discrimination, and orientation.^6^ Numbering among these instruments is the VOSP (*Visual Object and Space Perception Battery*). This battery evaluates space and object perception and is based on the assumption that these perceptions are functionally independent.^6^ The subtests require very simple responses, each of which focuses on one component of visual perception while minimizing the involvement of other cognitive skills.^15^

The aim of this preliminary study was to evaluate visuospatial function in mild AD patients using the VOSP, and to assess sensitivity of the battery in a Brazilian sample.

## Methods

### Participants

We evaluated 20 patients (11 females) with a mean age of 74.45 (SD 5.98) years and 8.40 (SD 4.98) years of education, with probable mild AD according to the criteria of the National Institute of Neurological and Communicative Disorders and Stroke and Alzheimer’s Disease and Related Disorders Association (NINCDS-ADRDA)^16^ recommended by the Brazilian Academy of Neurology.^13^ These patients were recruited at outpatient cognitive units in São Paulo Hospital and Santa Marcelina Hospital. The control group comprised 17 healthy individuals (9 females) with a mean age of 68.24 (9.78) years and 12.06 (3.90) years of education, who fulfilled inclusion criteria: higher than the median scores for educational level on the Mini-Mental State Examination (MMSE),^17,18^ a score of less than or equal to 6 on the Geriatric Depression Scale (reduced version - 15 items)^19^ and a score of less than 2 on the Functional Activities Questionnaire.^20^

In both groups, subjects had to have more than one year of education, be more than 50 years old, and have no uncorrected visual deficit or uncontrolled systemic diseases. The AD group included patients who had been in use of a stable dose of antidepressant or/and cholinesterase inhibitors for at least two months.

This study was approved by the Research Ethics Committees of the Federal University of São Paulo and Santa Marcelina Hospital. All participants (controls or caregivers) agreed to take part in the study by signing an informed consent form.

This research constitutes a preliminary study being part of a Master’s degree study.

### Neuropsychological evaluation

Cognitive functions of all participants were evaluated using the following instruments:

#### Complex Figure Test^21^ (Rey, 1983)

This instrument assesses perceptual organization and visual memory. First the patient has to copy the complex figure. After completion, an immediate recall task is performed, and after 30 minutes, a delayed recall of the complex figure.

#### Corsi Block-tapping Test^22^

The patient is asked to repeat (or replicate in reverse) the prearranged sequence that the examiner taps on the blocks. Evaluates attention (direct form) and working memory (inverse form).

#### Rey Auditory Verbal Learning Test (RAVLT)^23^

A series of word tests that assess verbal learning, memory and susceptibility to interference.

#### Verbal Fluency – animal category^24^

Participant states as many animals as possible in 60 seconds. This instrument evaluates spontaneous production of words under restricted conditions.^6^

#### Brief version of the Boston Naming Test – CERAD Neuropsychological Battery^25^

15 drawings from the Boston Naming Test are presented and the patient has to name them. This test evaluates visual naming ability.

#### Cancellation task^26^

Subjects are asked to mark predetermined target stimulus on a sheet of paper. We scored corrected and uncorrected signaled stimuli, as well as time taken to perform the task. The activity is interrupted after 300s.^27^

#### Raven’s Progressive Matrices – colored version^28^

A measure of intellectual efficiency and a visuoperception test in versions developed for children and older subjects. Test items require the examinee to infer a rule from a sequence of stimuli.

#### Clock Drawing Test^29^

Evaluates visuospatial and constructional abilities. The subject is asked to draw a clock face displaying the time 2:45.

### Visual Object and Space Perception Battery (VOSP)^15^

The evaluation of visuospatial abilities was carried out using the eight- test VOSP battery, four assessing perception of object and four assessing perception of space. Initially, a test of visual sensory efficiency (Shape Detection) is performed to check whether the patient has adapted visual and sensorial capacity for the others subtests. All tests are untimed.

#### Screening test

*Shape detection* – Patient has to identify whether there is an embedded “X” or otherwise on 20 all-over pattern sheets. One point is given for each correct answer. Subjects scoring 15 or lower are deemed unable to complete the VOSP battery.

#### Object perception subtests

*Incomplete letters* – 20 incomplete letters are presented and the patient is asked to identify them.

***Silhouettes*** – 15 silhouettes of animals and 15 silhouettes of objects are shown and the patient has to identify them.

*Object decision* – 20 sheets containing four stimuli are shown, and the patient has to decide which one represents the silhouette of a real object. Only one drawing depicts something real, while the others are distracter items.

*Progressive silhouettes* – Two series of silhouettes of two objects are presented and the patient has to identify them as quickly as possible. In the beginning objects are hard to identify, but become progressively easier as the examiner turns the sheets.

#### Space perception subtests

*Dot counting* – Patient has to count how many black dots there are on each of ten cards.

*Position discrimination* – 20 cards are presented, each of them containing two identical squares, both with a black dot on them. The patient has to identify in which of the squares the black dot is exactly in the center.

*Number location* – Ten cards are presented, each of containing two squares, one above the other. The top square has the numbers one to nine randomly distributed within it, and the other square has only a black dot. The patient has to identify which number corresponds exactly to the position of the black dot.

*Cube analysis* – Patient has to count how many blocks appear in a three-dimensional drawing on each card. Ten sheets are presented.

### Statistical analysis

Data analysis was performed using the SPSS 13.0 software. The demographic variables were analyzed from a descriptive viewpoint. Non-parametric tests were used to compare neuropsychological result variables and non-continuous variables (Mann-Whitney). The chosen significance level was 5% (p<0.05). The data were also analyzed by Spearman’s Correlation Test.

## Results

The demographic and clinical data is shown in Table 1. There was no difference between patients and control group regarding gender. However, differences were found between the two groups in relation to age and education level, where patients were older and controls had more education. All participants of the study had GDS scores of less than or equal to 6, showing no depressive symptoms. As expected, a significant difference between the two groups on the MMSE and the Functional Activities Questionnaire was observed.

**Table 1:** Demographic and clinical features of AD patients and healthy elderly controls.

	Diagnosis		Mann Whitney Testp- value
	Controls (n=17) Mean (SD)	Mild AD patients (n=20) Mean (SD)	
Age	68.24 (9.78)	74.45(5.98)	0.03
Educational level	12.06 (3.90)	8.40(4.98)	0.02
MMSE	28.35 (1.73)	24.25 (3.11)	<0.01
Pfeffer	0.18 (0.53)	11.05 (4.95)	<0.01
GDS	2.35 (1.58)	2.95 (1.88)	0.43

Performance on neuropsychological tests by AD patients and healthy controls is presented in Table 2. A significant difference was detected between groups on almost all tests, except the Corsi Block-Tapping test – reverse order (p=0.20), and on the cancellation task considering errors (p=0.141).

**Table 2:** Scores on neuropsychological evaluations in AD patient and control groups.

	Controls Mean (SD)	Probable AD Mean (SD)	Mann Whitney Test p-value
RAVLT - Total	46.76 (7.33)	23.85 (5.58)	<0.01
RAVLT - after interference	9.53 (2.83)	1.75 (2.24)	<0.01
RAVLT - after 30 minutes	8.65 (2.62)	0.85 (1.50)	<0.01
Raven - colored version	28.71 (6.00)	20.16 (4.96)	<0.01
Verbal fluency - animals	17.65 (4.24)	11.00 (3.51)	<0.01
Rey Complex Figure - copy	33.35 (2.50)	26.70 (6.98)	<0.01
Rey Complex Figure - immediate recall	19.29 (6.07)	6.00 (5.44)	<0.01
Rey Complex Figure - delayed recall	17.88 (7.22)	4.28 (6.30)	<0.01
Clock drawing test	8.59 (2.24)	6.65 (2.52)	<0.01
Corsi - direct (span)	5.12 (0.78)	3.90 (1.07)	<0.01
Corsi - inverse (span)	4.47 (1.12)	3.85 (1.23)	0.20
Boston naming (15 items)	14.59 (0.62)	12.35 (1.79)	<0.01
Cancellation task (number correct)	51.76 (8.52)	40.80 (12.73)	<0.01
Cancellation task (number of errors)	0.41 (0.71)	2.45 (4.17)	0.141
Cancellation task (time - seconds)	154.94 (47.16)	229.85 (48.91)	<0.01

Comparison of the scores of AD patients against those of controls on the visuospatial evaluation (VOSP) is shown in Table 3. None of the participants failed on the screening test, thus all patients and controls were eligible to perform the other subtests. No significant difference was observed between the results of the two groups on the Shape Detection Test, whereas a significant difference was detected on all four object perception tests, with the most significant differences being found on the Incomplete Letters, Silhouettes and Object Decision tests (p<0.01).

**Table 3:** Comparison between AD patients and healthy elderly on the VOSP.

	Controls Mean (SD)	Probable AD Mean (SD)	Mann Whitney Test p-value
Shape detection - screening test	19.29 (0.77)	18.60 (1.39)	0.12
Incomplete letters	19.41 (1.28)	17.25 (2.99)	<0.01
Silhouettes	19.06 (4.49)	12.90 (5.10)	<0.01
Object decision	16.35 (2.94)	12.60 (3.66)	<0.01
Progressive silhouettes	11.82 (2.79)	13.95 (2.86)	0.04
Dot counting	9.94 (0.24)	9.65 (0.59)	0.09
Position discrimination	19.41 (1.18)	18.80 (1.44)	0.08
Number location	8.65 (2.00)	6.80 (2.65)	0.01
Cube analysis	8.88 (1.76)	6.70 (2.77)	0.02

The scores on the space perception subtests revealed no significant difference between the groups on the Dot Counting (p=0.09) and Position Discrimination (p=0.08) tests. However, significant differences were detected on the Number Location (p=0.01) and Cube Analyses (p=0.02) tests.

Spearman’s Correlation test was used to investigate association among tests, and the Raven test was found to correlate with the VOSP screening test (r=0.511), both of which evaluate intellectual efficiency. The Boston Naming Test correlated with all object perception subtests of the VOSP: Incomplete Letters (r=0.536), Silhouettes (r=0.671), Object decision (r=0.654), and Progressive Silhouettes (r=0.591). On the space perception subtests, the Number Location was found to correlate with the Cancellation task (number correct) (r=0.501), and also with the Corsi Block tapping (direct) (r=0.640) test.

## Discussion

The aim of the present study was to evaluate all cognitive functions (visual and working memory, attention, verbal learning, language, naming ability, and intellectual efficiency, constructional and visuospatial ability) and to assess the global functioning of all participants. Akin to many previous reports by other authors,^4,5,30^ our AD patients showed worse results than control subjects on virtually all neuropsychological tests, except the Corsi Block – tapping (reverse) test which evaluates working memory. Working memory seems to remain relatively intact in mild AD patients.^5^ Similarly, no difference was observed in the cancellation tasks for errors.

AD patients performed poorly on the instrument that evaluates intellectual efficiency (Raven) where this correlated with the screening test of the VOSP. The Raven test has an important component of visual perception, besides measuring intellectual efficiency.

The cancellation task (number correct) was also shown to be performed poorly by AD patients, probably due to inattention and impairment in visuoperceptual function. This instrument was correlated with the VOSP subtest Number Location, which also requires attention and dealing with distracter stimuli. The Corsi Block – tapping (direct) test, which evaluates attention, also correlated with this VOSP subtest. These results support the importance of the attentional network in the perception of visuospatial stimuli.^8^

A significant impairment in the perception of objects was found, evidenced by the results on the Incomplete Letters, Silhouettes, Object Decision and Progressive Silhouettes tests. These scores were highly correlated with Boston Naming scores, on which patients with AD performed worse than controls, as expected.^31^ This is consistent with the assumption that AD patients are impaired on visual-perceptual tasks such as visual object recognition and figure/ground discrimination.^9,11,14,32^

On the Boston Naming Test, the majority of errors among AD patients (71.4%) were because the subject was unable to name the picture (omission). This kind of error suggests that one possible cause of object recognition impairment in AD could be a deficit in processing structural aspects of visually presented items, and not only a deficit in the semantic system.^33^

Among the spatial perception tests carried out, only the Number location and Cube analysis showed differences between AD patients and the healthy elderly group. The Dot Counting and Position Discrimination showed no significant difference.

Assuming that the task of identifying and locating objects employs different cortical areas, these results could be due to the different cortical areas affected.^6^ Visuospatial tasks can be mediated by either dorsal or ventral visual processing neural streams. These streams are distinct neural circuits that project from the striate cortex to the posterior parietal (dorsal) or inferotemporal (ventral) cortices. The dorsal pathway analyzes spatial aspects and motion, while the ventral analyzes form and color information.^34^

The differences in age and educational level detected among the AD patients and control subjects could have influenced the results presented in this preliminary study since it is known that differences between subjects with lower education (e.g. between a subject with 1 year and another with 3 years of education) are more significant than between individuals with higher educational levels.

However, our findings support the assumption that visual tasks are valuable for diagnosing AD and that cognitive deficits of this patient group could stem from visual-perceptual problems.^9^

Several subtests of the VOSP proved effective for detecting visuospatial impairment in mild AD patients, according to the results on the other neuropsychological tests. Further studies on a larger sample, matched for age and educational level, are now needed to confirm our preliminary findings.
